# Baseline Hemoglobin and Outcomes in Advanced Chronic Kidney Disease with Moderate-to-Severe Myocardial Ischemia: A Secondary Analysis of the ISCHEMIA-CKD Trial

**DOI:** 10.3390/jcm15218610

**Published:** 2026-11-05

**Authors:** Anar Mammadli, Erdem Diker

**Affiliations:** 1 Department of Cardiology, Acıbadem Bayındır Söğütözü Hospital, Ankara 06250, Türkiye; 2 Department of Cardiology, School of Medicine, Ankara Medipol University, Ankara 06050, Türkiye; erdem.diker@ankaramedipol.edu.tr

**Keywords:** anemia, chronic kidney disease, hemoglobin, myocardial ischemia, prognosis

## Abstract

**Background/Objectives:** Hemoglobin may identify differences in prognosis among patients with advanced chronic kidney disease (CKD) and documented myocardial ischemia. We examined the shape of its association with clinical outcomes in ISCHEMIA-CKD. **Methods:** This exploratory observational secondary analysis included 758 randomized participants with baseline hemoglobin. The primary endpoint was the parent-trial composite of all-cause death or myocardial infarction (MI). Adjusted Cox regression with restricted cubic splines characterized the hemoglobin association. Mortality and pooled nonfatal MI were examined using Cox and competing-risk models. **Results:** Median hemoglobin was 11.1 g/dL; 247 participants experienced the primary endpoint. The adjusted association departed from log-linearity (*p* = 0.015), with higher hazard at lower hemoglobin and an uncertain upper limb. Relative to 11.1 g/dL, the hazard ratio (HR) at 8 g/dL was 1.98 (95% confidence interval [CI] 1.26–3.10). As a linear summary, the HR per 1 g/dL lower hemoglobin was 1.13 (95% CI 1.04–1.23) for the primary endpoint. Corresponding mortality associations were present, whereas pooled nonfatal MI showed no clear association (subdistribution HR 1.03, 95% CI 0.89–1.21). Exploratory treatment interaction tests provided no convincing evidence of effect modification (linear *p* = 0.059; spline *p* = 0.124). **Conclusions:** Among selected, clinically stable participants with advanced CKD and moderate-to-severe ischemia, lower baseline hemoglobin was associated with adverse prognosis. Nonlinearity and uncertainty at higher values argue against a single summary estimate or an inferred optimal target. These potentially confounded findings neither establish benefit from hemoglobin correction nor support hemoglobin-based selection of invasive versus conservative management.

## 1. Introduction

Patients with chronic kidney disease (CKD) and coronary artery disease (CAD) have substantial cardiovascular morbidity and mortality [1]. Patients with advanced CKD have also been under-represented in trials of coronary management [2]. ISCHEMIA-CKD addressed this population by randomizing 777 participants with estimated glomerular filtration rate (eGFR) < 30 mL/min/1.73 m^2^ or dialysis dependence, and at least moderate ischemia on stress testing, to initial invasive or conservative management. The invasive strategy did not reduce the trial’s primary endpoint of death or myocardial infarction (MI) [3]. The availability of adjudicated outcomes in this population provides an opportunity to examine baseline characteristics associated with subsequent prognosis.

Anemia commonly accompanies CKD, and observational studies have associated lower hemoglobin with adverse cardiovascular outcomes and mortality [4,5,6]. Coronary-intervention registries have also reported higher risk in patients with both anemia and renal dysfunction [7,8]. However, these observations arise from populations that differ in kidney disease severity, clinical presentation and treatment. Their relevance to patients with advanced CKD and objectively documented ischemia, therefore, requires careful interpretation. Hemoglobin may also have a nonlinear relationship with outcomes, making a single coefficient or binary anemia threshold an incomplete description of its prognostic association.

We evaluated baseline hemoglobin in the ISCHEMIA-CKD cohort, emphasizing the shape and uncertainty of its association with all-cause death or MI. Secondary objectives were to examine all-cause death, cardiovascular death and pooled nonfatal MI, and to explore whether the association of randomized treatment strategy with the primary endpoint varied according to hemoglobin.

## 2. Materials and Methods

This was an exploratory observational secondary analysis of individual participant data from ISCHEMIA-CKD. The trial’s design, eligibility criteria and primary results have been published previously [3,9]. Of 777 randomized participants, 758 with an available baseline hemoglobin measurement were included. In the parent trial, median follow-up was 2.2 years (interquartile range, 1.6–3.0), and 12 participants (1.5%) withdrew or were lost to follow-up [3].

Participants were randomized 1:1 to an initial invasive or an initial conservative coronary management strategy, both with optimal medical therapy. The invasive strategy added catheterization, targeted within 30 days of randomization, and revascularization by percutaneous coronary intervention (PCI) or coronary artery bypass grafting as appropriate, with a hydration protocol and contrast-volume limits for participants not receiving dialysis, and with transradial access recommended to limit bleeding [3,9]. In the conservative strategy, catheterization was reserved for failure of medical therapy, such as an acute coronary syndrome or refractory angina [3,9]. Anemia management was not protocol-specified in either strategy and followed local practice in line with kidney disease guidelines [9]; no anemia-treatment data were available for this analysis (Section 2.2).

Individual participant data were obtained from the National Heart, Lung, and Blood Institute through the Biologic Specimen and Data Repository Information Coordinating Center under a data-use agreement. The parent trial received ethics approval at participating centers, and participants provided informed consent [3]. The present secondary analysis was approved by the Scientific, Medical Ethics and Deontology Board of Acıbadem Bayındır Söğütözü Hospital, Ankara (approval no. ABTEDK-06/26).

### 2.1. Exposure and Outcomes

The exposure was baseline hemoglobin (g/dL), measured within the 6 months before randomization. Sampling dates, timing relative to dialysis, acute illness or treatment, and follow-up hemoglobin values were not available. Hemoglobin was retained as a continuous variable, to preserve information. For descriptive tables and supplementary categorical analyses, anemia was classified using sex-specific thresholds of <13 g/dL in men and <12 g/dL in women [10]. WHO adult cutoffs were applied uniformly across the cohort, for descriptive purposes. Severity categories were severe (<8 g/dL), moderate (8 to <11 g/dL), mild (11 to <13 g/dL in men or 11 to <12 g/dL in women), and non-anemic.

The primary endpoint was the supplied parent-trial composite of all-cause death or MI, using the primary MI definition. Secondary endpoints were all-cause death, cardiovascular death and pooled nonfatal MI. Pooled nonfatal MI denotes the first adjudicated nonfatal MI under the primary MI definition [3], with MI types analyzed together. We used the repository’s adjudicated endpoint-specific event indicators and times. For nonfatal MI, all-cause death before nonfatal MI during that endpoint’s observation period was treated as a competing event; for cardiovascular death, non-cardiovascular death was the competing event. Endpoint-specific follow-up was retained because mortality observation could extend beyond the last assessment for nonfatal events. Consequently, a later death contributing to the primary endpoint did not necessarily appear as a competing death in the nonfatal-MI analysis.

### 2.2. Covariates and Missing Data

The core adjustment set comprised age, sex, dialysis status, eGFR among participants not receiving dialysis, left ventricular ejection fraction (LVEF), diabetes and randomized treatment arm. These variables represent demographic characteristics, kidney disease severity, ventricular function, diabetes and randomized management strategy; their selection was informed by the parent-trial analysis and published ancillary analyses [3,11,12,13]. Age, non-dialysis eGFR, and LVEF were modeled with three-knot restricted cubic splines at the 10th, 50th and 90th percentiles. For eGFR spline construction, dialysis participants were assigned the non-dialysis median, with a separate dialysis indicator retained. Covariates were limited to trial-defined baseline variables and the randomized arm; no clinical variables recorded during follow-up were included.

We used the trial-supplied imputed LVEF variable; no additional imputation was performed for this secondary analysis. Baseline hemoglobin and all covariates in the core adjustment set were complete in the 758 included participants. To assess sensitivity to additional measured characteristics, an expanded-adjustment model added body mass index, current smoking, hypertension, previous MI, history of heart failure, ischemia severity, and baseline use of statins, aspirin, an angiotensin-converting enzyme inhibitor or angiotensin receptor blocker, and beta-blockers. Body mass index was modeled with a three-knot spline at the 10th, 50th and 90th percentiles of available values in the analytic cohort. Both linear and spline hemoglobin specifications were evaluated with expanded adjustment. This model used 743 complete cases with 238 primary-endpoint events; core-adjusted models were also refitted in these complete cases. Missingness is described in Supplementary Table S1. Neither adjustment set included erythropoiesis-stimulating agent (ESA) or iron therapy, iron status, transfusion history, blood loss, specific inflammatory markers (e.g., C-reactive protein), serum albumin, direct measures of volume status (e.g., natriuretic peptides) or frailty measures, none of which was available in the repository dataset.

### 2.3. Statistical Analysis

Continuous baseline characteristics were summarized as medians and interquartile ranges, and categorical characteristics as counts and percentages. Cox proportional hazards regression was used for the primary endpoint. Hemoglobin was modeled with a four-knot restricted cubic spline at the 5th, 35th, 65th and 95th percentiles of its cohort distribution. Departure from log-linearity was assessed by a likelihood-ratio test comparing spline and linear specifications. The nonlinear curve was emphasized in interpretation, with the cohort median hemoglobin of 11.1 g/dL as reference. Pointwise 95% confidence intervals (CIs) for hazard ratios (HRs) were calculated from the difference between the model design vectors at each hemoglobin value and the reference, using the fitted coefficient covariance matrix.

A linear hemoglobin term, coded per 1 g/dL lower hemoglobin, was also fitted in separate models (hereafter, linear summary models) to provide a concise summary. For the primary endpoint, that coefficient was not interpreted as a constant association across the observed range after evidence of nonlinearity. All-cause death was modeled with Cox regression. Cardiovascular death and pooled nonfatal MI were modeled with Fine–Gray subdistribution hazard regression and complementary cause-specific Cox regression. The latter censored competing deaths at their recorded times. Fine–Gray models used expanded risk sets with the corresponding weights and participant-clustered robust variance estimates. These secondary models retained linear hemoglobin terms, and therefore summarize associations under that functional-form assumption.

Exploratory hemoglobin-by-treatment interactions were assessed using both linear and four-knot spline specifications and likelihood-ratio tests. Conditional invasive-versus-conservative HRs were derived from the corresponding fitted coefficients and covariance matrices. Supplementary analyses also included descriptive subgroup comparisons, categorical anemia analyses and the parent trial’s key secondary composite of death, MI, hospitalization for unstable angina or heart failure, or resuscitated cardiac arrest. The primary analysis was the core-adjusted spline model of the primary endpoint and its likelihood-ratio test of nonlinearity. Models of the secondary endpoints and the exploratory treatment-interaction analyses addressed the secondary objectives; subgroup analyses were also exploratory, and all other analyses were supportive. No adjustment for multiplicity was applied to *p*-values or CIs; all *p*-values were two-sided and nominal, and were interpreted alongside point estimates, 95% CIs and model assumptions. The proportional hazards assumption for core and expanded Cox models of the primary endpoint was assessed using scaled Schoenfeld residuals. Analyses were performed using R 4.6.1.

## 3. Results

The analytic cohort included 758 participants, representing 97.6% of those randomized; 19 were excluded because baseline hemoglobin was unavailable. Median hemoglobin was 11.1 g/dL (interquartile range, 10.1–12.4), and 603 participants (79.6%) met the anemia definition. There were 155 non-anemic participants, 262 with mild anemia, 318 with moderate anemia and 23 with severe anemia. Only 46 participants (6.1%) had hemoglobin > 14 g/dL. Baseline characteristics are summarized in Table 1.

### 3.1. Primary Endpoint and Association Shape

**Figure 1:**
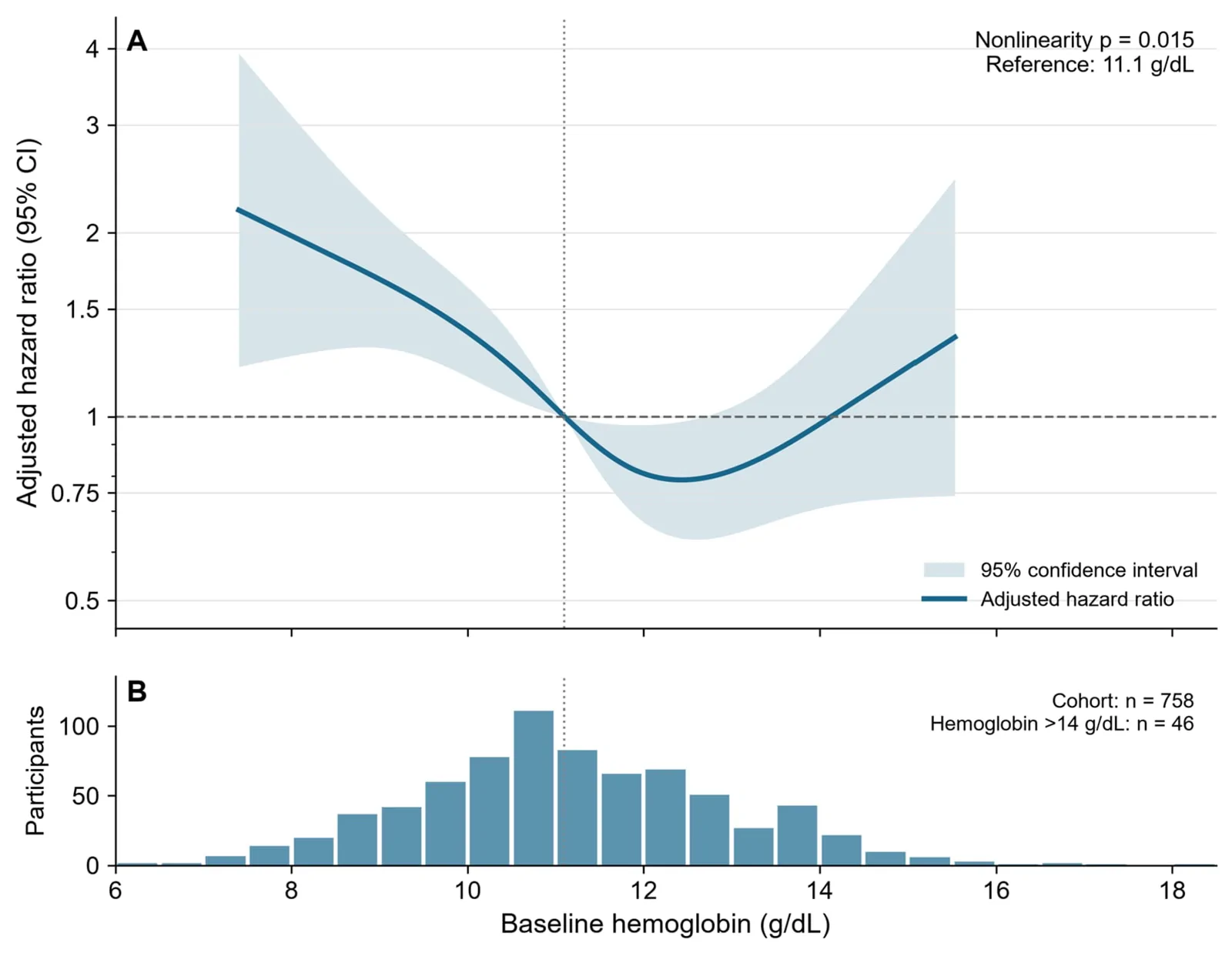
Adjusted association between baseline hemoglobin and the primary endpoint of all-cause death or myocardial infarction. (**A**) Four-knot restricted cubic spline, referenced to hemoglobin 11.1 g/dL. The shaded band is the pointwise 95% confidence interval calculated for the contrast against that reference; it has zero width at the reference. Knots are at 8.4, 10.6, 11.8 and 14.2 g/dL. The curve is displayed across the 1st–99th percentiles (7.4–15.5 g/dL). Core adjustment is as specified in Table 2. (**B**) The histogram shows the entire analytic cohort, including observations outside the displayed spline range. The fitted minimum is descriptive, and does not identify a treatment target.

The primary endpoint occurred in 247 participants. At 12, 24 and 36 months, 637, 388 and 169 participants remained at risk (Supplementary Figure S1); this decline mainly reflected events and administrative censoring at the end of trial follow-up, rather than dropout. The spline model showed evidence of departure from log-linearity (likelihood-ratio *p* = 0.015). Relative to hemoglobin 11.1 g/dL, the adjusted HR was 1.98 (95% CI 1.26–3.10) at 8 g/dL and 1.20 (95% CI 0.74–1.96) at 15 g/dL; contrasts at other hemoglobin values, including after expanded adjustment, are shown in Supplementary Table S3. The fitted curve suggested higher hazard at lower hemoglobin, with less precise estimation in the upper tail. Its minimum occurred near 12.4 g/dL, but this data-derived location was not estimated as a clinical threshold or treatment target. The apparent upper limb remained uncertain, given the small number of participants at higher hemoglobin values (Figure 1).

**Table 2:** Associations of baseline hemoglobin with clinical outcomes in linear summary models.

Outcome	Model	Events	HR or sHR (95% CI)	*p*
Primary endpoint (all-cause death or MI)	Cox	247	1.13 (1.04–1.23)	0.004
All-cause death	Cox	188	1.13 (1.03–1.25)	0.011
Cardiovascular death	Fine–Gray	155	1.14 (1.02–1.27)	0.020
Cardiovascular death	Cause-specific Cox	155	1.14 (1.03–1.27)	0.013
Pooled nonfatal MI	Fine–Gray	83	1.03 (0.89–1.21)	0.660
Pooled nonfatal MI	Cause-specific Cox	83	1.07 (0.92–1.23)	0.382

All models include 758 participants. Estimates are per 1 g/dL lower baseline hemoglobin from linear summary models. The primary association is nonlinear, so its linear HR should not be read as a constant association across the hemoglobin range; Figure 1 shows the fitted shape. Core adjustment: age, sex, dialysis status, eGFR among non-dialysis participants, trial-imputed LVEF, diabetes and randomized arm. Age, non-dialysis eGFR and LVEF use three-knot restricted cubic splines. For cardiovascular death, 33 non-cardiovascular deaths were competing events. For nonfatal MI, 142 deaths were competing events during nonfatal-event observation. A further 22 deaths after that observation ended contribute to mortality and the primary endpoint. CI, confidence interval; HR, hazard ratio; MI, myocardial infarction; sHR, subdistribution hazard ratio. All *p*-values are nominal.

The linear summary model yielded an adjusted HR of 1.13 (95% CI 1.04–1.23; *p* = 0.004) per 1 g/dL lower hemoglobin. This is a model-dependent summary, rather than a uniform association across the hemoglobin range. Scaled Schoenfeld residual tests did not identify evidence against proportional hazards for the core linear (global *p* = 0.939) or spline (global *p* = 0.971) model. In the expanded-adjustment complete-case analysis, the corresponding linear HR was 1.15 (95% CI 1.05–1.25; *p* = 0.002). The spline specification also retained evidence of nonlinearity after expanded adjustment (*p* = 0.037; with core adjustment in the same complete cases, *p* = 0.041). Full results for these models are provided in Supplementary Table S2.

### 3.2. Mortality and Nonfatal Myocardial Infarction

There were 188 all-cause deaths. The adjusted linear Cox HR per 1 g/dL lower hemoglobin was 1.13 (95% CI 1.03–1.25; *p* = 0.011). Cardiovascular death occurred in 155 participants, with 33 non-cardiovascular deaths treated as competing events. The corresponding Fine–Gray subdistribution HR was 1.14 (95% CI 1.02–1.27; *p* = 0.020), and the cause-specific Cox HR was 1.14 (95% CI 1.03–1.27; *p* = 0.013). These estimates are summarized in Table 2.

Pooled nonfatal MI occurred in 83 participants, with 142 deaths recorded as competing events during nonfatal-MI observation. The linear hemoglobin association was imprecisely estimated, and showed no clear signal: subdistribution HR was 1.03 (95% CI 0.89–1.21; *p* = 0.66) and cause-specific HR 1.07 (95% CI 0.92–1.23; *p* = 0.38). A further 22 participants died after their last MI/unstable-angina assessment. They were censored at that earlier assessment for nonfatal MI, while their later deaths contributed to the primary and mortality endpoints. This difference in endpoint-specific observation accounts for the difference between 247 primary-endpoint events and the 225 nonfatal-MI events plus competing deaths.

### 3.3. Exploratory and Supplementary Analyses

Treatment interaction tests did not provide convincing evidence that baseline hemoglobin modified the relative effect of initial invasive versus conservative management (linear interaction *p* = 0.059; spline interaction *p* = 0.124; Supplementary Table S4). Conditional treatment estimates were imprecise, and sensitive to model specification. For example, at 8 g/dL, the invasive-versus-conservative HR was 0.66 (95% CI 0.39–1.12) in the linear interaction model, but 1.35 (95% CI 0.64–2.85) in the spline interaction model; all 95% CIs from 8 to 13 g/dL included 1 (Supplementary Table S5).

Across subgroups defined by dialysis status, sex, diabetes and LVEF < 45%, primary-endpoint HRs ranged from 1.03 to 1.29 per 1 g/dL lower hemoglobin, without clear interaction (smallest *p* = 0.060, for sex; Supplementary Table S6). Relative to non-anemic participants, adjusted HRs were 0.86 (95% CI 0.58–1.27), 1.41 (95% CI 0.97–2.04) and 2.39 (95% CI 1.11–5.16) for mild, moderate and severe anemia, although only 23 participants (nine events) had severe anemia (Supplementary Table S7). For the parent trial’s key secondary composite (264 events), the linear summary HR was 1.15 (95% CI 1.06–1.25; *p* < 0.001; Supplementary Table S8).

## 4. Discussion

In this secondary analysis of ISCHEMIA-CKD, baseline hemoglobin was associated with the risk of death or MI after adjustment for measured baseline characteristics. The relationship was nonlinear, with higher hazard at lower hemoglobin and considerable uncertainty at the upper end of the distribution. Linear summary models also showed associations with all-cause and cardiovascular death, whereas the estimate for pooled nonfatal MI was less precise, and compatible with no association. These findings characterize prognosis within a selected trial population with advanced CKD and documented ischemia.

This selection limits external validity. ISCHEMIA-CKD enrolled clinically stable patients with eGFR < 30 mL/min/1.73 m^2^ or dialysis dependence and at least moderate ischemia on site-interpreted stress testing [3,9]. Exclusions included LVEF < 35%, New York Heart Association class III–IV heart failure or hospitalization for heart failure within 6 months, acute coronary syndrome within 2 months, refractory or rapidly progressive angina, high bleeding risk precluding dual antiplatelet therapy, and limited life expectancy due to non-cardiovascular comorbidity [9]. The present findings may therefore not apply to patients with less advanced CKD, without documented moderate-to-severe ischemia, with acute coronary syndromes, or with advanced heart failure or LVEF < 35%.

The mortality findings are consistent with population-based and CKD studies linking anemia to adverse outcomes [4,5,6]. Studies in coronary-intervention populations similarly identify patients with both anemia and renal dysfunction as a group with higher event rates [7,8,14]. A meta-analysis of PCI studies reported an association between lower hemoglobin and mortality, although differences in population, endpoints and effect measures limit direct quantitative comparison with the present estimates [15]. The PRAISE registry also examined the prognostic value of a hemoglobin-to-creatinine ratio in ST-elevation MI [16]. Its acute presentation and ratio-based exposure differ from baseline hemoglobin in this advanced-CKD cohort. The contribution of the present study is therefore the characterization of association shape and uncertainty in the specific ISCHEMIA-CKD population, rather than a new causal explanation for anemia-related risk.

The nonlinear model is important to the interpretation of the primary result. A single linear HR can obscure variation across the hemoglobin range, and should not imply that every 1 g/dL difference has the same association with outcome. Although the fitted curve turned upward at higher values, evidence for a reproducible high-hemoglobin risk increase was limited by sparse observations and wide CIs. A nonlinear curve can also have an apparent minimum without identifying a clinically meaningful threshold. The value near 12.4 g/dL should, therefore, not be described as an optimum or used to guide treatment. A reported U-shaped association in a large hemodialysis registry provides context, but does not independently validate the upper limb or minimum in this smaller cohort [17].

Hemoglobin in advanced CKD reflects multiple clinical processes, and may have different prognostic implications, depending on how a given concentration was reached. Because iron deficiency, inflammation, nutritional status, blood loss and ESA or iron therapy could not be assessed directly in the repository dataset (Section 2.2), the observed associations may partly reflect these factors, rather than hemoglobin concentration itself (Limitations Section). Observational prognostic associations cannot determine whether raising hemoglobin would improve outcomes. Trials of ESA-based correction toward near-normal hemoglobin or hematocrit targets have not established cardiovascular benefit, and have raised safety concerns [18,19,20]. The distinction between a measured risk marker and an intervention target is therefore essential when interpreting the present results.

The absence of a clear association with pooled nonfatal MI should also be interpreted cautiously. This endpoint had fewer events than mortality, and its CI remained compatible with both lower and higher hazard. Furthermore, nonfatal-event assessment and vital-status follow-up did not always end at the same time. Retaining the supplied endpoint-specific censoring preserves the distinction between these observation processes, but does not remove possible bias if loss of nonfatal-event observation is related to unmeasured health status. If nonfatal-event observation ended earlier among sicker participants with lower hemoglobin, the nonfatal-MI association could be underestimated, and the MI component of the primary endpoint is subject to the same concern. These results should not be read as evidence that hemoglobin has no relationship with nonfatal MI.

The exploratory treatment analyses did not provide a reliable basis for selecting between invasive and conservative strategies according to hemoglobin. A nonsignificant interaction test does not establish equivalence of treatment effects across all hemoglobin values, particularly when conditional estimates are imprecise. Conversely, uncertainty does not justify a claim of differential treatment benefit. The current results should be interpreted alongside the randomized treatment comparison in the parent trial [3]. Baseline hemoglobin may contribute to clinical recognition of a higher-risk phenotype, but this study did not assess whether it improves discrimination, calibration or decision-making, beyond existing prognostic information.

### Limitations

Several limitations constrain interpretation. Hemoglobin was an observational exposure, and its association with outcomes does not inherit the causal protection of randomization. The most important limitation is, therefore, residual confounding. Iron deficiency, inflammation, nutritional status, volume status, blood loss, ESA therapy, transfusion, frailty and other disease processes can influence both hemoglobin and prognosis, and none of the specific factors listed was measured directly in the repository dataset (Section 2.2). Because inflammation, a recognized cardiovascular risk factor in CKD [1], and hemodilution from volume overload can both lower hemoglobin and accompany higher risk, the adjusted association may overstate any contribution of hemoglobin itself, whereas ESA therapy and transfusion, which raise hemoglobin but are given mainly to sicker patients, could bias it in either direction. Persistence of an association after expanded adjustment does not establish absence of residual confounding.

Only a single baseline hemoglobin value was available. Within-person variability, including fluctuation with dialysis timing and volume shifts, would attenuate the association toward the null (regression dilution), whereas values obtained during acute illness might accentuate it; without repeated measurements, neither effect could be quantified. Hemoglobin may also have changed after baseline through anemia treatment, bleeding or kidney disease progression, including dialysis initiation in 18% of parent-trial participants not receiving dialysis at baseline [13]. The estimates, therefore, describe the prognosis associated with a single baseline value, not with hemoglobin over time or with changing it.

Beyond the trial-level selection discussed in Section 4, analysis-specific choices also warrant caution. Participants with missing baseline hemoglobin were excluded, and the expanded model required complete cases. Although the excluded proportions were small, these choices do not guarantee freedom from selection bias. Trial-supplied LVEF imputation was retained; uncertainty arising from that imputation was not separately modeled in the present analysis.

The relatively small upper-hemoglobin group limits estimation of curve shape in that region. Pointwise confidence bands do not provide uncertainty for the location of the apparent minimum or establish a simultaneous confidence region for the entire curve. Linear summary models of the secondary endpoints may conceal endpoint-specific nonlinearity. Multiple exploratory analyses were performed without multiplicity correction, and interaction estimates were insufficiently precise for clinical treatment selection. No external validation or incremental prediction assessment was undertaken. These findings require confirmation in other appropriately characterized advanced-CKD populations.

## 5. Conclusions

Among selected, clinically stable ISCHEMIA-CKD participants, lower baseline hemoglobin was associated with adverse prognosis after adjustment for measured covariates. The primary association was nonlinear, with an uncertain high-hemoglobin limb. Lower hemoglobin was associated with mortality; the pooled nonfatal-MI estimate remained imprecise. These exploratory observational findings may partly reflect residual confounding by unmeasured anemia-related factors. They do not identify an optimal hemoglobin concentration, establish benefit from correction, or support hemoglobin-based selection of invasive versus conservative management.

## Figures and Tables

**Table 1:** Baseline characteristics overall and by WHO anemia severity.

Characteristic	Overall (*n* = 758)	Non-Anemic (*n* = 155)	Mild (*n* = 262)	Moderate (*n* = 318)	Severe (*n* = 23)
Age, years	63.0 [56.0–70.0]	66.0 [58.0–74.0]	64.0 [57.0–70.0]	62.0 [55.0–69.0]	53.0 [46.5–65.0]
Male sex	518/758 (68.3)	96/155 (61.9)	196/262 (74.8)	212/318 (66.7)	14/23 (60.9)
Female sex	240/758 (31.7)	59/155 (38.1)	66/262 (25.2)	106/318 (33.3)	9/23 (39.1)
Race					
Black or African American	62/728 (8.5)	2/149 (1.3)	20/260 (7.7)	35/299 (11.7)	5/20 (25.0)
Other	200/728 (27.5)	16/149 (10.7)	69/260 (26.5)	105/299 (35.1)	10/20 (50.0)
White	466/728 (64.0)	131/149 (87.9)	171/260 (65.8)	159/299 (53.2)	5/20 (25.0)
Body mass index, kg/m^2^	27.0 [23.0–31.0]	27.0 [24.0–31.0]	27.0 [23.0–30.0]	26.0 [23.0–31.0]	27.0 [23.0–30.0]
Hemoglobin, g/dL	11.1 [10.1–12.4]	13.7 [13.0–14.2]	11.7 [11.2–12.3]	10.0 [9.3–10.5]	7.6 [7.2–7.8]
Dialysis-dependent	401/758 (52.9)	50/155 (32.3)	140/262 (53.4)	197/318 (61.9)	14/23 (60.9)
eGFR among non-dialysis participants, mL/min/1.73 m^2^	23.0 [17.2–26.8]	25.7 [22.9–28.1]	23.2 [17.9–26.9]	18.8 [14.7–24.0]	10.2 [8.8–14.8]
LVEF, %	60.0 [50.0–63.0]	58.0 [50.0–62.5]	60.0 [50.0–64.0]	58.5 [49.5–62.9]	61.6 [55.0–63.0]
LVEF < 45%	88/758 (11.6)	18/155 (11.6)	33/262 (12.6)	33/318 (10.4)	4/23 (17.4)
Diabetes mellitus	430/758 (56.7)	74/155 (47.7)	137/262 (52.3)	204/318 (64.2)	15/23 (65.2)
Hypertension	695/755 (92.1)	142/154 (92.2)	244/262 (93.1)	289/316 (91.5)	20/23 (87.0)
Prior myocardial infarction	131/757 (17.3)	37/155 (23.9)	38/262 (14.5)	52/317 (16.4)	4/23 (17.4)
History of heart failure	194/758 (25.6)	46/155 (29.7)	63/262 (24.0)	78/318 (24.5)	7/23 (30.4)
Current smoker	87/758 (11.5)	23/155 (14.8)	26/262 (9.9)	36/318 (11.3)	2/23 (8.7)
Moderate ischemia	461/750 (61.5)	98/151 (64.9)	160/260 (61.5)	190/316 (60.1)	13/23 (56.5)
Severe ischemia	289/750 (38.5)	53/151 (35.1)	100/260 (38.5)	126/316 (39.9)	10/23 (43.5)
Statin use	618/758 (81.5)	125/155 (80.6)	218/262 (83.2)	257/318 (80.8)	18/23 (78.3)
Aspirin use	565/758 (74.5)	119/155 (76.8)	193/262 (73.7)	240/318 (75.5)	13/23 (56.5)
ACEi/ARB use	364/758 (48.0)	82/155 (52.9)	131/262 (50.0)	146/318 (45.9)	5/23 (21.7)
Beta-blocker use	568/758 (74.9)	123/155 (79.4)	188/262 (71.8)	239/318 (75.2)	18/23 (78.3)
Randomized invasive strategy	379/758 (50.0)	78/155 (50.3)	138/262 (52.7)	151/318 (47.5)	12/23 (52.2)

Values are median [interquartile range] or *n*/N (%), where N is the number with available data. In this table, hemoglobin categories are descriptive; regression models use continuous hemoglobin, except the categorical analysis (Supplementary Table S7). Severe anemia: <8 g/dL; moderate: 8 to <11 g/dL; mild: 11 to <13 g/dL in men and 11 to <12 g/dL in women; non-anemic: ≥13 g/dL in men and ≥12 g/dL in women. eGFR summaries concern non-dialysis participants only (overall *n* = 357; non-anemic *n* = 105; mild *n* = 122; moderate *n* = 121; severe *n* = 9). Body mass index is missing in three participants (one mild, two moderate); categorical denominators display their own missingness. LVEF uses the trial-supplied imputed variable. ACEi/ARB, angiotensin-converting enzyme inhibitor/angiotensin receptor blocker; eGFR, estimated glomerular filtration rate; LVEF, left ventricular ejection fraction; WHO, World Health Organization.

## Data Availability

The data are available to qualified investigators upon application to the National Heart, Lung, and Blood Institute through the Biologic Specimen and Data Repository Information Coordinating Center (BioLINCC).

## Supplementary Materials

The following supporting information can be downloaded at: https://www.mdpi.com/article/10.3390/jcm15218610/s1. Table S1: Missingness of covariates for expanded adjustment; Table S2: Expanded-adjustment sensitivity analyses; Table S3: Selected contrasts from the nonlinear hemoglobin models; Table S4: Exploratory hemoglobin-by-treatment interaction tests; Table S5: Conditional invasive-versus-conservative treatment estimates; Table S6: Exploratory subgroup associations with the primary endpoint; Table S7: Categorical analysis by WHO anemia severity; Table S8: Additional composite endpoint; Figure S1: Kaplan–Meier estimates by WHO anemia severity.

## Abbreviations

ACEi/ARB	Angiotensin-converting enzyme inhibitor/angiotensin receptor blocker
BioLINCC	Biologic Specimen and Data Repository Information Coordinating Center
CAD	Coronary artery disease
CKD	Chronic kidney disease
eGFR	Estimated glomerular filtration rate
ESA	Erythropoiesis-stimulating agent
LVEF	Left ventricular ejection fraction
MI	Myocardial infarction
NHLBI	National Heart, Lung, and Blood Institute
PCI	Percutaneous coronary intervention
sHR	Subdistribution hazard ratio
WHO	World Health Organization
