# Supplementary material for: Baseline Hemoglobin and Outcomes in Advanced Chronic Kidney Disease with Moderate-to-Severe Myocardial Ischemia: A Secondary Analysis of the ISCHEMIA-CKD Trial
Source: J Clin Med. 2026 Nov 5;15(21):8610. doi: 10.3390/jcm15218610 (PMC13764150; doi:10.3390/jcm15218610)
Supplement: Supplementary file 1 [file jcm-15-08610-s001.zip › Supplementary Appendix.pdf]

SUPPLEMENTARY APPENDIX

Baseline Hemoglobin and Outcomes in Advanced Chronic Kidney Disease with Moderate-to-Severe Myocardial Ischemia: A Secondary Analysis of the ISCHEMIA-CKD Trial

Anar Mammadli, Erdem Diker

CONTENTS

Table S1. Missingness of covariates for expanded adjustment

Table S2. Expanded-adjustment sensitivity analyses

Table S3. Selected contrasts from the nonlinear hemoglobin models

Table S4. Exploratory hemoglobin-by-treatment interaction tests

Table S5. Conditional invasive-versus-conservative treatment estimates

Table S6. Exploratory subgroup associations with the primary endpoint

Table S7. Categorical analysis by WHO anemia severity

Table S8. Additional composite endpoint

Figure S1. Kaplan–Meier estimates by WHO anemia severity

Abbreviations

ACEi/ARB	Angiotensin-converting enzyme inhibitor/angiotensin receptor blocker
CI	Confidence interval
CKD	Chronic kidney disease
eGFR	Estimated glomerular filtration rate
HR	Hazard ratio
LRT	Likelihood-ratio test
LVEF	Left ventricular ejection fraction
MI	Myocardial infarction
RCS	Restricted cubic splines
WHO	World Health Organization

Table S1. Missingness of covariates for expanded adjustment

Covariate	Missing, n	Missing, %
Age	0	0.0
Sex	0	0.0
Dialysis status	0	0.0
eGFR	0	0.0
LVEF (trial-imputed)	0	0.0
Diabetes	0	0.0
Randomized arm	0	0.0
Body mass index	3	0.4
Current smoking	0	0.0
Hypertension	3	0.4
Prior MI	1	0.1
History of heart failure	0	0.0
Ischemia severity	8	1.1
Statin use	0	0.0
Aspirin use	0	0.0
ACEi/ARB use	0	0.0
Beta-blocker use	0	0.0

Denominator: 758 participants with baseline hemoglobin available. Fifteen participants have at least one missing expanded-model covariate, leaving 743 complete cases with 238 primary events. Trial-supplied LVEF imputation was retained; no additional imputation was performed. The 19 randomized participants without baseline hemoglobin are outside this table.

Table S2. Expanded-adjustment sensitivity analyses

Model	n	Events	Linear HR (95% CI)	p
Core adjustment full cohort	758	247	1.13 (1.04–1.23)	0.004
Core adjustment complete cases	743	238	1.14 (1.04–1.24)	0.003
Expanded adjustment complete cases	743	238	1.15 (1.05–1.25)	0.002

Linear estimates are per 1 g/dL lower hemoglobin. Core adjustment comprises age, sex, dialysis, non-dialysis eGFR, LVEF, diabetes and randomized arm. Age, eGFR and LVEF use three-knot restricted cubic splines. Expanded adjustment additionally includes body mass index (three-knot spline at 21, 27 and 35 kg/m^2), current smoking, hypertension, prior MI, history of heart failure, ischemia severity and baseline statin, aspirin, ACEi/ARB and beta-blocker use. Linear models contain 11 core or 22 expanded regression parameters.

Likelihood-ratio tests for hemoglobin nonlinearity (two degrees of freedom): core full cohort $p = 0.015$; core complete cases $p = 0.041$; expanded complete cases $p = 0.037$. Global proportional-hazards tests: core linear $p = 0.939$, core spline $p = 0.971$, expanded linear $p = 0.888$ and expanded spline $p = 0.922$.

Table S3. Selected contrasts from the nonlinear hemoglobin models

Hemoglobin, g/dL	Core HR (95% CI) n = 758	Expanded HR (95% CI) n = 743
8	1.98 (1.26–3.10)	2.11 (1.32–3.37)
10	1.37 (1.16–1.63)	1.36 (1.14–1.62)
11.1 (Reference)	-	-
12	0.81 (0.67–0.97)	0.82 (0.68–0.99)
12.4	0.79 (0.63–0.98)	0.80 (0.64–1.00)
14	0.97 (0.71–1.33)	0.92 (0.66–1.29)
15	1.20 (0.74–1.96)	1.08 (0.64–1.81)

Table S4. Exploratory hemoglobin-by-treatment interaction tests

Specification	Interaction df	LRT χ^2	p
Linear Hemoglobin by treatment arm	1	3.557	0.059
Four-knot RCS Hemoglobin by treatment arm	3	5.756	0.124

Both comparisons use 758 participants and 247 primary events. The linear comparison adds one hemoglobin-by-arm term; the spline comparison adds three interaction terms to a model with spline hemoglobin main effects. The adjustment covariates are the core set described in Table S2.

Table S5. Conditional invasive-versus-conservative treatment estimates

Hemoglobin, g/dL	Linear interaction HR (95% CI)	Spline interaction HR (95% CI)
8	0.66 (0.39–1.12)	1.35 (0.64–2.85)
9	0.77 (0.51–1.14)	0.94 (0.62–1.43)
10	0.89 (0.66–1.19)	0.76 (0.51–1.12)
11	1.03 (0.80–1.33)	0.85 (0.61–1.20)
12	1.19 (0.88–1.61)	1.22 (0.75–1.98)
13	1.38 (0.93–2.06)	1.55 (0.94–2.55)

HR compares initial invasive with initial conservative management. Conditional estimates are derived from the respective model coefficients and covariance matrices.

Table S6. Exploratory subgroup associations with the primary endpoint

Subgroup	n	Events	Linear HR (95% CI)	Interaction p
Dialysis: No	357	104	1.15 (1.03–1.29)	0.808
Dialysis: Yes	401	143	1.13 (1.01–1.27)	0.808
Sex: Male	518	182	1.09 (0.99–1.19)	0.060
Sex: Female	240	65	1.29 (1.10–1.52)	0.060
Diabetes: No	328	79	1.03 (0.89–1.18)	0.100
Diabetes: Yes	430	168	1.18 (1.07–1.30)	0.100
LVEF <45%: No	670	206	1.14 (1.04–1.25)	0.844
LVEF <45%: Yes	88	41	1.12 (0.94–1.33)	0.844

HRs are per 1 g/dL lower hemoglobin. Within-subgroup estimates and interaction tests come from the same model for each stratifier. The hemoglobin main effect and interaction are linear; therefore, these analyses are descriptive summaries and do not capture the full nonlinear primary association.

Sex and diabetes analyses retain the core adjustment set, including the stratifier main effect. The dialysis analysis adjusts for age, sex, LVEF, diabetes and randomized arm, plus dialysis and its hemoglobin interaction; eGFR is omitted. The LVEF <45% analysis adjusts for age, sex, dialysis, non-dialysis eGFR, diabetes and randomized arm; the binary LVEF indicator and interaction replace continuous LVEF. Age, eGFR and LVEF measures use the same three-knot splines as the main model.

Table S7. Categorical analysis by WHO anemia severity

Anemia severity	n	Events	HR (95% CI)	p
Non-anemic	155	43	1.00 (reference)	—
Mild	262	67	0.86 (0.58–1.27)	0.450
Moderate	318	128	1.41 (0.97–2.04)	0.071
Severe	23	9	2.39 (1.11–5.16)	0.026

Main endpoint: death or MI (758 participants, 247 events). Adjustment uses the core set, with spline age, non-dialysis eGFR and LVEF. The severe category contains only 23 participants and nine events; its estimate is imprecise.

Table S8. Additional composite endpoint

Endpoint	n	Events	Linear HR (95% CI)	p
Death, MI, hospitalization for unstable angina or heart failure, or resuscitated cardiac arrest	758	264	1.15 (1.06–1.25)	<0.001

HR is per 1 g/dL lower hemoglobin, adjusted for the core covariate set.

Figure S1. Kaplan–Meier estimates by WHO anemia severity

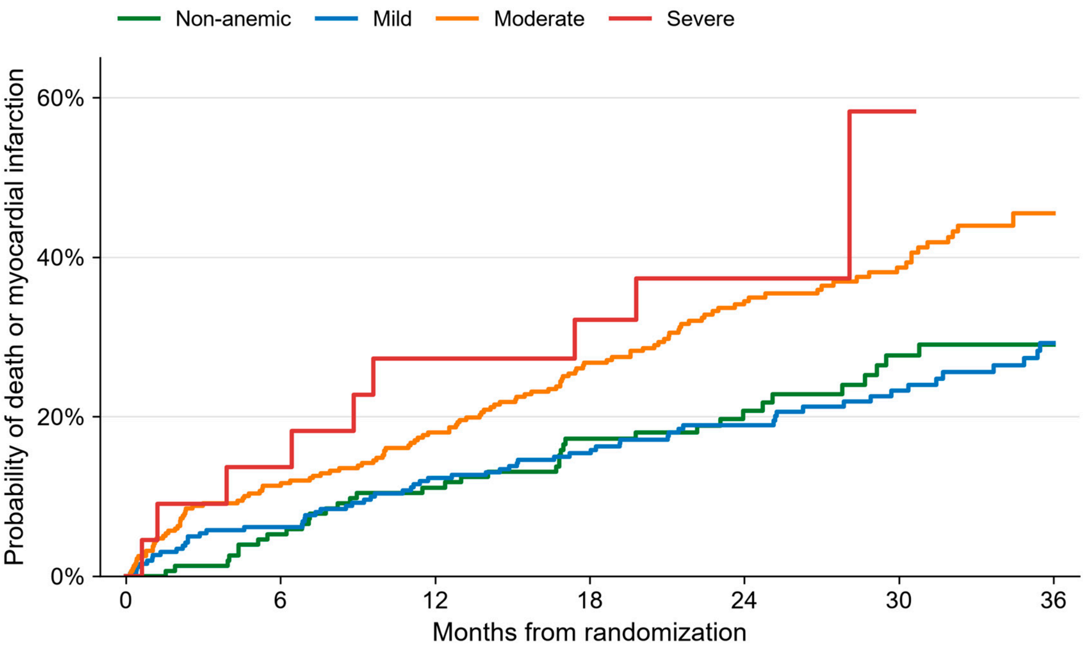

Number at risk

	0	6	12	18	24	30	36
Non-anemic	155	145	136	113	79	58	42
Mild	262	245	228	204	152	107	73
Moderate	318	281	257	212	149	100	54
Severe	23	19	16	14	8	1	0

Unadjusted probability of the primary composite of all-cause death or MI, estimated as one minus Kaplan–Meier survival. Categories are defined in Table 1. The number-at-risk table is shown below the curves.
